# Application of fundamental equations to species−area theory

**DOI:** 10.1186/s12898-016-0097-5

**Published:** 2016-10-07

**Authors:** Xubin Pan

**Affiliations:** Institute of Plant Quarantine, Chinese Academy of Inspection and Quarantine, Beijing, 100029 China

**Keywords:** Endemics-area relationship, Overlap-area relationship, Power law, Random replacement, Real total area, Sampling rate, Overlap rate, Extinction rate, Overlap index

## Abstract

**Background:**

Species−area relationship (SAR), endemics-area relationship (EAR) and overlap-area relationship (OAR) are three important concepts in biodiversity study. The application of fundamental equations linking the SAR, EAR and OAR, can enrich the axiomatic framework of the species−area theory and deepen our understanding of the mechanisms of community assembly.

**Results:**

Two fundamental equations are derived and extended to power law model and random replacement model of species−area distribution. Several important parameters, including the overlap index and extinction rate, are defined and expressed to enrich the species−area theory. For power law model, both EAR and OAR have three parameters, with one more parameter of the total area than SAR does. The EAR equation is a monotonically increasing function for parameter *c* and *z*, and a monotonically decreasing function for parameter *A*. The extinction rate, with two parameters, is a monotonically increasing function for parameter *z*, and a monotonically decreasing function for parameter *A*. The overlap index is a monotonically increasing function for parameter *A*, and a monotonically decreasing function for parameter *z*, independent of parameter *c*.

**Conclusions:**

The general formats of SAR, EAR, OAR, overlap index, overlap rate, sampling rate and extinction rate, are derived and extended to power law model and random replacement model as the axiomatic framework of species−area theory. In addition, if the total area is underestimated, the extinction rate will be overestimated.

**Electronic supplementary material:**

The online version of this article (doi:10.1186/s12898-016-0097-5) contains supplementary material, which is available to authorized users.

## Background

Species−area relationship (SAR) is a core concept in biodiversity and species distribution [1], and endemics-area relationship (EAR) is a useful tool in biodiversity conservation and habitat preservation [2–5]. Besides SAR and EAR, overlap-area relationship (OAR), which refers to the number of overlap species in two areas, is also a relevant and important concept [6–8]. To link SAR and EAR and develop a complete species−area theory, two fundamental equations are established to describe species distribution and interrelation between two compensatory areas [7]. Now the species−area theory has been reconstructed by the set theory, integrating SAR, EAR, OAR, alpha diversity, beta diversity, and gamma diversity [8]. Although OAR curves for two areas of the same size are described and zeta diversity as the average number of species shared by multi-assemblages is proposed, the expanding concept that compares two or more areas of different sizes has not been fully discussed yet [8, 9]. Furthermore, to investigate the spatial characteristics of species richness, it is necessary to integrate the two fundamental equations into the species−area model with distribution information or assumption. Then more parameters can be defined and expressed with empirical data, which can enrich the axiomatic framework of the species−area theory and deepen our understanding of the mechanisms or processes of community assembly.

In addition, debate still exists over the estimation of extinction rate based on the SAR, which is higher than observed extinction rate [10–13]. One explanation for the overestimation is that some species are “committed to extinction” instead of going extinct due to habitat clearing [14–16]. However, another reason has been ignored in this debate [8]. According to the power law model, the SAR is a two-parameter equation, whereas the EAR is a three-parameter equation. It does not consider total area in SAR, while total area and its corresponding total species number are crucial factors to determine species disappearing and extinction rate. However, the impact of total area on the extinction rate is still unknown without the specific species−area model and sensitivity analysis.

In this paper, power law and random replacement functions, both of which are widely used species−area models, were selected for the application of two fundamental equations [17–19]. Then several important parameters were defined and expressed to enrich the species−area theory. For power law model, sensitivity analysis of parameters was conducted for EAR, extinction rate and overlap index, and the extinction rate based on different total areas were assessed for overestimate comparison.

## Methods

**Fig. 1:**
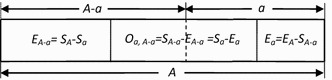
Schematic diagram of species distribution in Area (*a*) and Area (*A*−*a*) [7]

The relationships among SAR, EAR and OAR have been shown in Fig. 1, where *S*_*a*_ is the number of species in area *a*, *E*_*a*_ is the number of species that will disappear when habitat area *a* is cleared, *O*_*a, A*−*a*_ is the number of overlap species in two areas *a* and *A *− *a*,

1 $$\quad S_{A} \left( { = E_{A} } \right)$$

is the number of total species in the total area *A*. These relationships can be connected by two fundamental equations [7]:

2 $$S_{a} \; + \;E_{A - a} \; = \;S_{A} ,$$

and

3 $$O_{a, A - a} = S_{a} - E_{a}.$$

These fundamental Eqs. (2 and 3) for species−area theory were applied to power law model and random replacement model of SAR. To enrich the species−area theory, several parameters were proposed, including overlap index, overlap rate, sampling rate and extinction rate, which were defined by equations in the general format, power law model and random replacement model.

For power law model, sensitivity analysis of parameters was conducted for EAR, extinction rate and overlap index, and the extinction rate based on different total areas was assessed for overestimate comparison. The data can be downloaded from the Supplementary of Data.

## Results

### Application of two fundamental equations to power law model

Power law model has been widely used for species−area relationship:

4 $$S_{a} = ca^{z} ,$$

where *S*_*a*_ is the number of species in area *a*, and *c* (*a* = 1, *c* = *S*_*1*_) and *z* (0 ≤  z ≤1) are fitted constants [11, 17]. However, the corresponding function of endemics-area relationship (EAR) has not received much attention, such as in He and Hubbell’ paper [12],

5 $$S_{loss} \; = \;S_{A} - S_{A - a} \; = \;\;cA^{z} \; - \;c(A - a)^{z} ,$$

where S_*loss*_ is the number of species that disappear when habitat area *a* is cleared, *A* is the total area, and *S*_*A*_ is the total number of the species in area *A*. In fact,

6 $$E_{a} = S_{loss} = cA^{z} - c\left( {A - a} \right)^{z} ,$$

where *E*_*a*_ is the number of species that exist only in area *a*, but not in area *A* − *a*. Because

7 $${a + (A} - {a) = A, \quad S}_{a} { + }\;{E}_{A-a} { = ca}^{z} \;{ + }\;{cA}^{z} - \;{c(A} - ( {A} - {a))}^{z} { \,= \,cA}^{z} { = S}_{A} { = E}_{A}$$

(*E*_*A*_ is the total number of specific species in area *A*), another method to get EAR is to derive it based on the relationship between SAR and EAR. If area *A* −* a* is cleared while area *a* remains, the number of species that will disappear is *cA* ^*z*^ − *ca* ^*z*^. Thus, we can get the endemics-area curves,

8 $$E_{A - a} = cA^{z} - ca^{z}, \quad E_{a} = cA^{z} - c\left( {A - a} \right)^{z} ,$$

the same as those that are derived with the former method. SAR and EAR are rotationally symmetrical, with the center at (*A/2*, *S/2*) [7]. The OAR (*O*_*a, A*−*a*_) between the area *a* and *A* −* a* can be calculated as

9 $$\begin{aligned} O_{a, A - a} &= S_{a} + S_{A - a} - S_{A} \\ &= ca^{z} + c(A - a)^{z} - cA^{z} \\ &= S_{a} - E_{a} .\end{aligned}$$

**Fig. 2:**
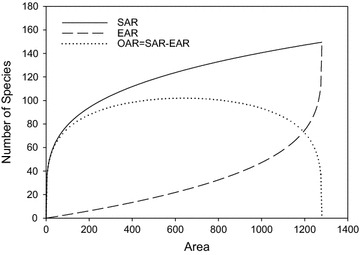
Species−area relationship (SAR), endemics-area relationship (EAR) and overlap-area relationship (OAR) (*A* = 1280, *c* = 25, and *z* = 0.25)

When it comes to *a* = *A*−*a*, *O*_*a, A*−__*a*_ attains its peak maximum value of $2{\text{c}}\left( {\frac{A}{2}} \right)^{z} - cA^{z}$. When *A* = 1280, *c* = 25, and *z* = 0.25, the SAR, EAR and OAR curves are shown in Fig. 2.

**Fig. 3:**
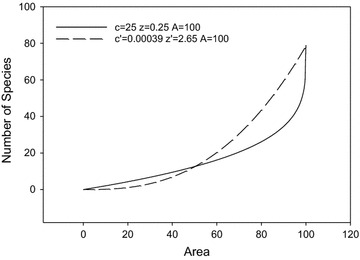
A comparison of the endemics-area relationship of this paper (*solid*) and that following power law format (*long dash*). $c^{'} = cA^{{(z - z^{')} }} ;1/2^{{z^{'} }} = 1 - 1/2^{z}$

For power law format of SAR, both EAR and OAR equations have three parameters: *c*, *z* and *A*. The EAR equation derived from power law function is different from the previous power law format of “endemics-area relationship”:

10 $$E_{a} = c\prime a^{{z\prime}} ,$$

where

11 $$c\prime = cA^{{(z - z^{\prime})}}$$

12 $$1/2^{{z^{\prime} }} = 1 - 1/2^{z}$$

which is based on the community-level fractal model [4, 12, 20]. A comparison of these two EAR curves is shown in Fig. 3, where three intersecting points are: (0, 0), (*A*/2, *cA* ^*z*^−*cA* ^*z*^/2^*z*^), and (*A*, *cA* ^*z*^). In the interval (0, *A*/2), power law format of “endemics-area relationship” based on fractal model underestimates the number of endemic species; in the interval (*A*/2, *A*), however, the power law format overestimates the number of endemic species. Both formats are three-parameter equations, but the latter equation is derived based on community-level fractal and power-law assumptions, which has decreased the accuracy of the model. Additionally, the former equation is simple and easy for parameter fitting. In the EAR curve, the species number decreases slowly at the beginning of habitat loss. Due to accumulation effect of habitat loss, the extinction rate of endemic species will speed up until all species disappear. Although the species extinction rate seems small at the beginning of land clearing, it will be too late to conserve biodiversity when most of the habitat disappears.

Overlap index *h* is proposed here,

13 $$h = \frac{{O_{a, A - a} }}{{S_{a} }} = \frac{{S_{a} - E_{a} }}{{S_{a} }} = 1 - \frac{{E_{a} }}{{S_{a} }}.$$

When *a* = *A*/2,* h* reaches 2 − 2^*z*^, the same value as obtained with bisection scheme [6]. We can get the overlap index for power law model as

14 $$\begin{aligned} h &= \frac{{O_{a, A - a} }}{{S_{a} }} \\ &= \frac{{ca^{z} + c(A - a)^{z} - cA^{z} }}{{ca^{z} }} \\ &= \frac{{a^{z} + (A - a)^{z} - A^{z} }}{{a^{z} }} \\ &= 1 + \left( {\frac{A}{a} - 1} \right)^{z} - \left( {\frac{A}{a}} \right)^{\text{z}} \\ &= 1 + \left( {\frac{1}{x} - 1} \right)^{\text{z}} - \left( {\frac{1}{x}} \right)^{\text{z}} , \end{aligned}$$

where $x = \frac{a}{A}$ is the ratio of area *a* over total area *A*. The overlap index *h* is the function of *z* and *x,* independent of *c*. Then parameter *z* reflects the overlapping or self-similarity properties of species number in power law function of SAR [6]. The ratio of overlapping species number over the total species number, overlap rate, is

15 $$h' = \frac{{O_{a, A - a} }}{{S_{A} }} = \frac{{S_{a} - E_{a} }}{{S_{A} }}.$$

Since *S*_*A*_ is constant,* h′* has a similar shape with *O*_*a, A*−*a*_, and will reach its peak value at 2^1−*z*^ − 1 when *a* = *A*/2. The ratio of *S*_*a*_ over *S*_*A*_, sampling rate, is

16 $$\eta = \frac{{S_{a} }}{{S_{A} }} = c\left( {\frac{a}{A}} \right)^{z} = cx^{z}.$$

The extinction rate is

17 $$\lambda = \frac{{E_{a} }}{{S_{A} }} = \frac{{cA^{z} - c\left( {A - a} \right)^{z} }}{{cA^{z} }} = 1 - (1 - x)^{z} ,$$

the format of which coincides with the previous extinction rate in the species−area curves [3].

### Application of two fundamental equations to random replacement model

Random replacement models for SAR and EAR are

18 $$S_{a} = S_{a}^{1} = S_{A} - \sum\limits_{i = 1}^{{S_{A} }} \left( {1 - \frac{a}{A}} \right)^{{N_{i} }} ,$$

19 $$E_{a} = S_{a}^{N} = \sum \limits_{i = 1}^{{S_{A} }} \left( {\frac{a}{A}} \right)^{{N_{i} }} ,$$

respectively, where *N*_*i*_ is the number of individuals of species *i*, and *S*_*a*_^*1*^ and *S*_*a*_^*N*^ are species−area curve and endemics-area curve across all species in *A*, respectively [12, 17].

20 $$\begin{aligned}S_{a} + E_{A - a} &= S_{A} - \sum\limits_{i = 1}^{S_{A}} {\left( 1 - \frac{a}{A} \right)^{N_{i}}} + \sum\limits_{i = 1}^{S_{A} } \left( \frac{A - a}{A} \right)^{N_{i}} \\ &= S_{A} = E_{A} ,\end{aligned}$$

corresponding with the fundamental equation.

21 $$\begin{aligned} O_{a, A - a} &= S_{a} - E_{a} \\ & = S_{A} - \sum \limits_{i = 1}^{S_{A}} \left( {1 - \frac{a}{A}} \right)^{N_{i} } - \sum \limits_{i = 1}^{S_{A}} (\frac{a}{A})^{N_{i}} \\ & = S_{A} - \sum \limits_{i = 1}^{S_{A}} \left[ \left( {1 - \frac{a}{A}} \right)^{N_{i}} + \left( {\frac{a}{A}} \right)^{N_{i} } \right] \end{aligned}$$

22 $$\begin{aligned} O_{a, A - a} &= S_{a} + S_{A - a} - S_{A} = S_{A} - \sum \limits_{i = 1}^{{S_{A} }} \left( {1 - \frac{a}{A}} \right)^{{N_{i} }} + S_{A} - \sum \limits_{i = 1}^{S_{A} } \left( \frac{a}{A} \right)^{N_{i}} - S_{A} \\ & = S_{A} - \sum \limits_{i = 1}^{S_{A}} \left[ {\left( 1 - \frac{a}{A} \right)^{N_{i} } + \left( \frac{a}{A} \right)^{N_{i} }} \right] \end{aligned}$$

23 $$\begin{aligned} h &= \frac{O_{a, A - a}}{S_{a} } = \frac{S_{a} - E_{a} }{S_{a} } \\ & = 1 - \frac{E_{a} }{S_{a} } \\ & = 1 - \frac{\sum\nolimits_{i = 1}^{S_{A} } \left( \frac{a}{A} \right)^{N_{i}} }{S_{A} - \sum \nolimits_{i = 1}^{S_{A} }\left( 1 - \frac{a}{A} \right)^{N_{i} } }\end{aligned}$$

24 $$\begin{aligned} h^{\prime} &= \frac{O_{a, A - a} }{S_{A} } = \frac{S_{a} + S_{A - a} - S_{A} }{S_{A} } \\ & = \frac{2S_{A} - \sum \nolimits_{i = 1}^{S_{A} } \left[ \left( 1 - \frac{a}{A} \right)^{N_{i}} + \left( \frac{a}{A} \right)^{N_{i} } \right]}{S_{A}} - 1\end{aligned}$$

When *a* = *A/2*, *O*_*a, A*−*a*_ and $h^{'}$ reaches its maximum value

25 $$O_{a, A - a} = S_{A} - 2 \sum \limits_{i = 1}^{{S_{A} }} \left( {\frac{1}{2}} \right)^{{N_{i} }}$$

26 $$\begin{aligned} h & = \frac{{2S_{A/2} - S_{A} }}{{S_{A/2} }} \\ & = 2 - \frac{{S_{A} }}{{S_{A/2} }} \\ & = 2 - \frac{{S_{A} }}{{S_{A} - \sum \nolimits_{i = 1}^{{S_{A} }} \left( {\frac{1}{2}} \right)^{{N_{i} }} }} \\ & = 2 - \frac{1}{{1 - \frac{{ \sum \nolimits_{i = 1}^{{S_{A} }} \left( {\frac{1}{2}} \right)^{{N_{i} }} }}{{S_{A} }}}} \end{aligned}$$

27 $$h^{'} = \frac{{ 2\left[ {S_{A} - \sum \nolimits_{i = 1}^{{S_{A} }} \left( {\frac{1}{2}} \right)^{{N_{i} }} } \right]}}{{S_{A} }} - 1$$

28 $$\eta = \frac{{S_{a} }}{{S_{A} }} = 1 - \frac{{E_{A - a} }}{{S_{A} }} = 1 - \frac{{ \sum \nolimits_{i = 1}^{{S_{A} }} \left( {1 - \frac{a}{A}} \right)^{{N_{i} }} }}{{S_{A} }}$$

29 $$\lambda = \frac{{E_{a} }}{{S_{A} }} = \frac{{ \sum \nolimits_{i = 1}^{{S_{A} }} \left( {\frac{a}{A}} \right)^{{N_{i} }} }}{{S_{A} }}$$

The general format, power law model and random replacement model for SAR, EAR and OAR are shown in Table 1.

**Table 1:** General format, power law and random placement models for SAR, EAR and OAR

Parameters	General format	Power law model	Random placement model
SAR EAR	*S*_*a*_ + *E*_*A*-*a*_ = *S*_*A*_ = *E*_*A*_ *S*_*A*-*a*_ + *E*_*a*_ = *S*_*A*_ = *E*_*A*_	*S*_*a*_ = *ca* ^*z*^ *E*_*a*_ = *cA* ^*z*^ − *c*(*A* − *a*)^*z*^	$S_{a} = S_{a}^{1} = S_{A} - \sum \limits_{i = 1}^{{S_{A} }} \left(1 - \frac{a}{A}\right)^{{N_{i} }}$
			$$E_{a} = S_{a}^{N} = \sum \limits_{i = 1}^{{S_{A} }} (\frac{a}{A})^{{N_{i} }}$$
OAR	$O_{a, A - a} = S_{a} - E_{a}$ $O_{a, A - a} = S_{a} + S_{A - a} - S_{A}$ $O_{a, A - a} = S_{A} - E_{a} - E_{A - a}$ $O_{a, A - a} = O_{A - a, a}$	$O_{a, A - a} = ca^{z} + c(A - a)^{z} - cA^{z}$	$O_{a, A - a} = S_{A} - \sum \limits_{i = 1}^{{S_{A} }} \left[ {\left( {1 - \frac{a}{A}} \right)^{{N_{i} }} + \left( {\frac{a}{A}} \right)^{{N_{i} }} } \right]$
*h*	$h = \frac{{O_{a, A - a} }}{{S_{a} }} = \frac{{S_{a} - E_{a} }}{{S_{a} }} = 1 - \frac{{E_{a} }}{{S_{a} }}$	$h = 1 - \frac{{A^{z} - (A - a)^{z} }}{{a^{z} }}$	$h = 1 - \frac{{ \sum \nolimits_{i = 1}^{{S_{A} }} \left(\frac{a}{A}\right)^{{N_{i} }} }}{{S_{A} - \sum \nolimits_{i = 1}^{{S_{A} }} \left(1 - \frac{a}{A}\right)^{{N_{i} }} }}$
*h, a* = *A*/2	$h = \frac{{2S_{A/2} - S_{A} }}{{S_{A/2} }} =$ *2*−$\frac{{S_{A} }}{{S_{A/2} }}$	*h* = 2 − 2^*z*^	$h = 2 - \frac{1}{{1 - \frac{{ \sum \nolimits_{i = 1}^{{S_{A} }} \left(\frac{1}{2}\right)^{{N_{i} }} }}{{S_{A} }}}}$
*h*′	$h^{'} = \frac{{O_{a, A - a} }}{{S_{A} }} = \frac{{S_{a} + S_{A - a} }}{{S_{A} }} - 1$	$h^{'} = \frac{{ca^{z} + c(A - a)^{z} }}{{cA^{z} }} - 1$	$h^{'} = \frac{{2S_{A} - \sum \nolimits_{i = 1}^{{S_{A} }} \left[ {\left( {1 - \frac{a}{A}} \right)^{{N_{i} }} + \left( {\frac{a}{A}} \right)^{{N_{i} }} } \right] }}{{S_{A} }} - 1$
*h*′, a = A/2	$h^{'} = \frac{{O_{a, A - a} }}{{S_{A} }} = \frac{{2S_{A/2} }}{{S_{A} }} - 1$	*h* ^’^ = 2^1−*z*^ − 1	
$\eta$	$\eta = \frac{{S_{a} }}{{S_{A} }} = 1 - \frac{{E_{A - a} }}{{S_{A} }}$	$\eta = c(\frac{a}{A})^{z}$	$\eta = 1 - \frac{{ \sum \nolimits_{i = 1}^{{S_{A} }} (1 - \frac{a}{A})^{{N_{i} }} }}{{S_{A} }}$
*λ*	$\lambda = \frac{{E_{a} }}{{S_{A} }} = 1 - \frac{{S_{A - a} }}{{S_{A} }}$ *λ* = (1 − *h*)*η*	$\lambda = 1 - c(1 - \frac{a}{A})^{z}$	$\lambda = \frac{{ \sum \nolimits_{i = 1}^{{S_{A} }} (\frac{a}{A})^{{N_{i} }} }}{{S_{A} }}$

*SAR* species−area relationship; *EAR* endemic-area relationship; *OAR* overlap-area relationship; *S*_*a*_ is the number of species in area *a*; *E*_*a*_ is the number of species only in the area *a*, but not in the area *A*-*a*; *O*_*a, A*−*a*_ is the number of species both in the area *a* and *A*−*a*; *c* (*a* = 1, *c* = *S*_*1*_) and *z* (0 ≤ z≤1) are constants; *A* is the total area, *S*_*A*_ is total number of the species in the area *A*, and *E*_*A*_ is the total number of specific species in area *A*; *h* is overlap index; *h*’ is the ratio of overlapping species number over the total species number, overlap rate; $\eta$ is ratio of *S*_*a*_ over *S*_*A*_, sampling rate; $\lambda$. is extinction rate; *N*_*i*_ is the number of individuals of the specific species *i*; *S*_*a*_^*1*^ and *S*_*a*_^*N*^ are species−area curve and endemics-area curve across all species in *A* [3, 6, 7, 10, 12, 16, 17]

### Sensitivity analysis for power law model

**Fig. 4:**
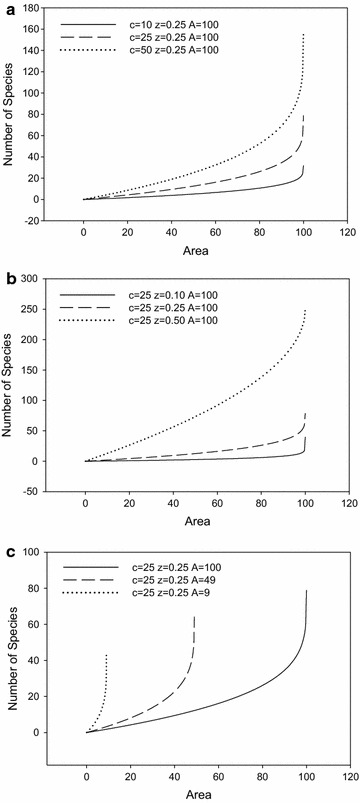
Sensitivity analysis of *c* (**a**), *z* (**b**), and *A* (**c**) on the power law format of endemics-area equation

The EAR equation is a monotonically increasing function for parameter *c*. In Fig. 4a, when *c* increases from 10 to 50, the number of extinct species increases from 32 to 158. The EAR equation is a monotonically increasing function for parameter *z*. In Fig. 4b, when *z* increases from 0.1 to 0.5, the number of extinct species increases from 40 to 250. The EAR equation is a monotonically decreasing function for parameter *A*. In Fig. 4c, when *A* increases from 9 to 100, the number of extinct species in the same area decreases, providing an important theoretical support for large habitat preservation.

**Fig. 5:**
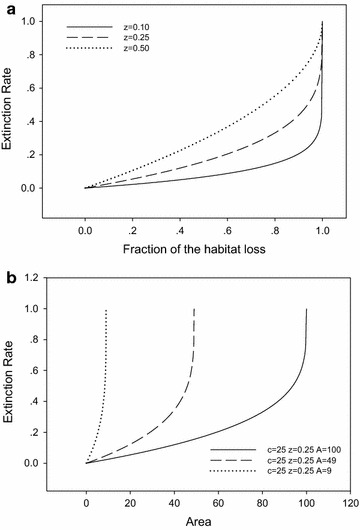
Sensitivity analysis of *z* (**a**) and *A* (**b**) on the extinction rate

Extinction rate is a monotonically increasing function for parameter *z* in the interval (0, 1). In Fig. 5a, when the fraction of habitat loss *x* increases from 0 to 1, the extinction rate also increases from 0 to 1. The extinction rate derived from EAR equation displays a similar pattern for parameter *A*. In Fig. 5b, when *A* increases from 9 to 100, the percentage of extinct species in the same area decreases.

**Fig. 6:**
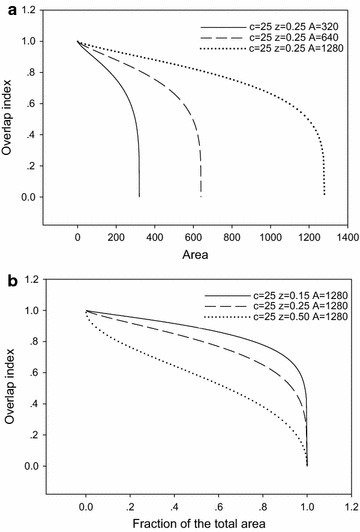
Sensitivity analysis of *A* (**a**) and *z* (**b**) on the overlap index

A sensitivity analysis of two parameters (*z* and *A*) is conducted for the overlap index *h*. The *h* equation is a monotonically increasing function for parameter *A* in Fig. 6a, when *A* increases from 320 to 1280. The *h* equation is a monotonically decreasing function for parameter *z* in Fig. 6b, when *z* increases from 0.15 to 0.5.

## Discussion

Table 2 shows the extinction rate estimate and overestimate comparison. If the real total area is 100, overestimate would occur when the total area is set to, say, 9 or 49. If the total area *A* is underestimated for 51 %, the overestimate of extinction rates will be 1.04 and 1.36 for 0.52 and 25 % of real total habitat loss, respectively [13, 21, 22]. However, the power law format of EAR will underestimate the extinction rate for the 0.52 and 25 % of real total habitat loss. If the total area *A* is underestimated for 91 %, the overestimate of extinction rates will be 10.34 and 40.91 for 0.52 and 9 % of real total habitat loss, respectively. Thus for field estimate of extinction rate, species−area relationship is an important tool, plus boundary identification of real total area, which can be assisted by remote sensing and geographic information system. If a smaller total area is adopted compared with the actual area, the species extinction rate estimate will be overstated.

**Table 2:** Extinction rate estimate and overestimate comparison

Area of habitat loss	*c* = 25 *z* = 0.25 *A* = 9		*c*= 25 *z*= 0.25 *A* = 49		*c*= 25 *z*= 0.25 *A* = 100
	λ (%)	Overestimate^a^	λ (%)	Overestimate^a^	λ_100_ (%)
0.52	1.48	10.34	0.27	1.04	0.13
1.00	2.90	10.56	0.51	1.05	0.25
9.00	100.00	41.91	4.95	1.12	2.33
25.00			16.34	1.36	6.94

^a^Overestimate = (λ−λ_100_)/λ_100_

Based on EAR, however, small total area for habitat preservation does lead to potential high species extinction rate. Thus large total area should be adopted for the Natural Protected Areas (NPAs). UNESCO-MAB World Network of Biosphere Reserves, suggests to apply a zonation system to NPAs, which consists of a core zone, a buffer zone and a transition zone. Normally, both the buffer zone and transition zone do not have any different or concerned species that are not in the core zone, thus the total number of species will not increase when the protected area is expanded from core zone to include the buffer zone and transition zone. But both the buffer zone and transition zone can relieve the impact of anthropic activities on the core zone, and this result can be derived from the species−area theory.

Since EAR and OAR involve species in two complementary areas, one more parameter, the total area, has been added in their expressions compared with SAR. If the concepts of EAR and OAR are expanded to arbitrary two areas (they can be treated as complementary in the point of mathematics), then the *h′* will be transferred to the Jaccard index, and Sørensen index can also be expressed by $\frac{{2O_{a, A - a} }}{{S_{A} + 2O_{a, A - a} }}$ [23–25]. If the concepts of EAR and OAR are expanded to more areas, then zeta diversity and new beta diversity can handle this circumstance [8, 9].

## Conclusions

Fundamental equations for species−area theory are applied to power law model and random replacement model of SAR. To enrich the species−area theory, several parameters are proposed, including overlap index, overlap rate, sampling rate and extinction rate, which are defined by equations in the general format, power law model and random replacement model. For power law model, both EAR and OAR have three parameters, with one more parameter of the total area than SAR does. If the total area is underestimated, the extinction rate will be overestimated. The EAR equation is a monotonically increasing function for parameter *c* and *z*, and a monotonically decreasing function for parameter *A*. Extinction rate, which has two parameters, is a monotonically increasing function for parameter *z*, and a monotonically decreasing function for parameter *A*. The overlap index is a monotonically increasing function for parameter *A*, and a monotonically decreasing function for parameter *z*, independent of parameter *c*.

## Additional file

10.1186/s12898-016-0097-5 For power law model, sensitivity analysis of parameters was conducted for EAR, extinction rate and overlap index, and the extinction rate based on different total areas was assessed for overestimate comparison.

## Abbreviations

SAR: species−area relationship
EAR: endemics-area relationship
OAR: overlap-area relationship
